# Fungal Garden Making inside Bamboos by a Non-Social Fungus-Growing Beetle

**DOI:** 10.1371/journal.pone.0079515

**Published:** 2013-11-05

**Authors:** Wataru Toki, Yukiko Takahashi, Katsumi Togashi

**Affiliations:** 1 Department of Forest Science, Graduate School of Agricultural and Life Sciences, the University of Tokyo, Bunkyo, Tokyo, Japan; 2 Department of Natural Environmental Studies, Graduate School of Frontier Sciences, the University of Tokyo, Kashiwa, Chiba, Japan; 3 Department of Forest Science, Graduate School of Agricultural and Life Sciences, the University of Tokyo, Bunkyo, Tokyo, Japan; University of Freiburg, Germany

## Abstract

In fungus-growing mutualism, it is indispensable for host animals to establish gardens of the symbiotic fungus as rapidly as possible. How to establish fungal gardens has been well-documented in social fungus-farming insects, whereas poorly documented in non-social fungus-farming insects. Here we report that the non-social, fungus-growing lizard beetle *Doubledaya bucculenta* (Coleoptera: Erotylidae: Languriinae) transmits the symbiotic yeast *Wickerhamomyces anomalus* from the ovipositor-associated mycangium into bamboo internode cavities and disperses the yeast in the cavities to make gardens. Microbial isolation and cryo-scanning electron microscopy observation revealed that *W. anomalus* was constantly located on the posterior ends of eggs, where larvae came out, and on the inner openings of oviposition holes. Direct observation of oviposition behavior inside internodes revealed that the distal parts of ovipositors showed a peristaltic movement when they were in contact with the posterior ends of eggs. Rearing experiments showed that *W. anomalus* was spread much more rapidly and widely on culture media and internodes in the presence of the larvae than in the absence. These results suggest that the ovipositors play a critical role in vertical transmission of *W. anomalus* and that the larvae contribute actively to the garden establishment, providing a novel case of fungal garden founding in non-social insect-fungus mutualism.

## Introduction

In a mutualism where a host animal depends obligately on a symbiont, it is critically important to manage and inherit the symbiont. Particularly, in a cultivation mutualism, where a host (farmer) cultures and consumes an ectosymbiont (crop, cultivar) as food, planting and proliferation as well as management of the symbionts are essential. At the highest level of cultivation mutualism observed in social insects such as ants, termites, and ambrosia beetles, the host adults directly promote the construction of a fungal garden by inoculation as well as reinoculation of suitable substrate with symbiotic fungi [1–6]. By contrast, in cultivation mutualism exhibited by non-social organisms such as a marine snail [7], several damselfish species [8], a slime mold [9], and several insects [10–15], we have poorly understood to what extent farmers contribute to garden making and management of cultivars.

Special structures for fungal transportation termed fungal pockets or mycangia have been found not only in social ants and ambrosia beetles exhibiting cultivation mutualism [16,17] but also in various non-social insects such as wood wasps [18], gall midges [19], lymexylid beetles [10,20], leaf-rolling weevils [11], lizard beetles [15], stag beetles [21], and others [22]. In plant-tissue boring insects among them, however, the process of introducing symbiotic fungi from mycangia into the substrates has been poorly investigated probably due to the difficulty of conducting direct observations inside plants. A well-documented case is the wood wasp *Sirex Noctilio* that possesses ovipositor-associated mycangia. Female wasps often bore two or more tunnels into the host tree trunks and deposit the eggs in the early made tunnels and the symbiotic fungus in the final tunnel separately through the ovipositor [23]. Coutts and Dolezal [23] suggest that deposition of eggs and fungi into different tunnels may prevent fungus-induced oleoresin from killing the wasp eggs. Thus, fungal inoculation is due to the ovipositor-associated mycangium directly onto the oviposition substrate.

**Figure 1:**
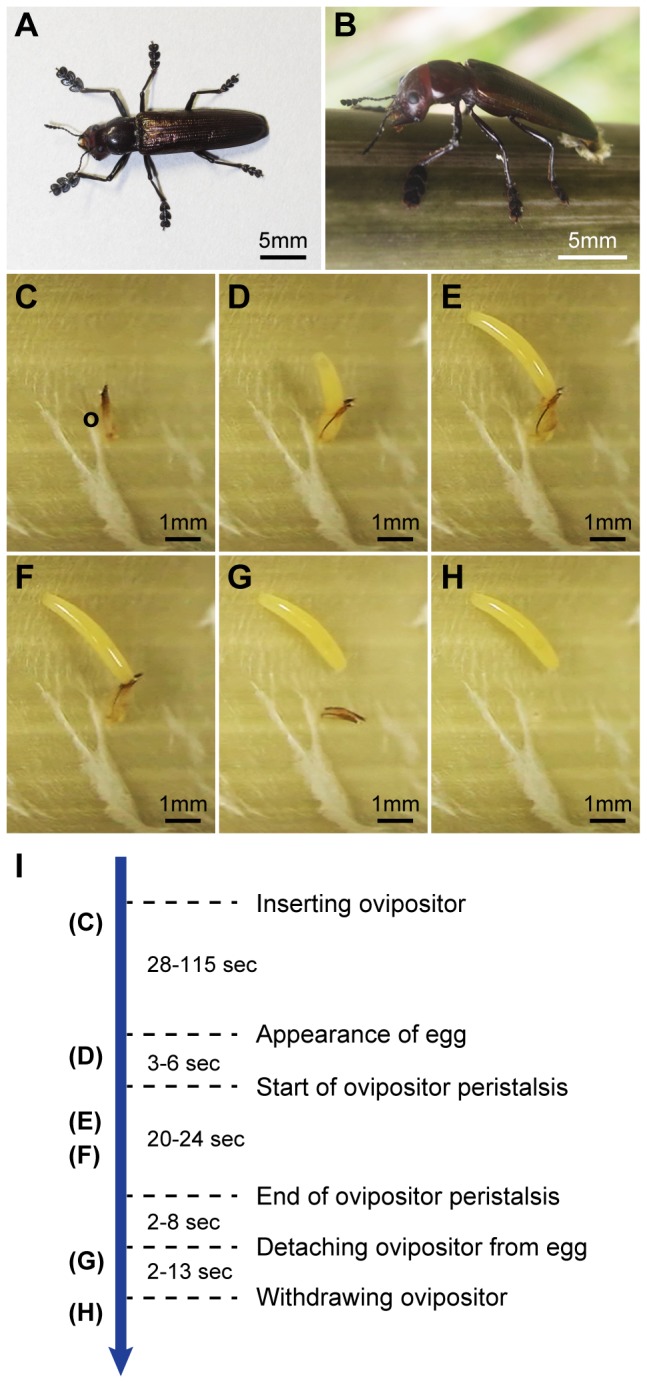
Female adult and behavioral sequence of egg deposition of *Doubledaya bucculenta*. (A) A female adult. (B) A female adult inserting the ovipositor into the internode cavity of *Pleioblastus simonii* for oviposition. (C to H) Behavioral sequence of *D. bucculenta* egg deposition. (C) An ovipositor being inserted into the internode cavity via the oviposition hole the female made. (D) An egg coming out of the ovipositor. The anterior end of banana-shaped egg is shown. (E, F) Inflated (E) and deflated (F) states of the distal part of ovipositor. Before the ovipositor is detached from the egg, it showed peristaltic movement. (G) Egg detached from the ovipositor completely. (H) Egg left behind on the inner surface of internode cavity. Immediately after depositing an egg, she pulled out the ovipositor. (I) Time course of behavioral events in oviposition. Capitals in parentheses correspond to the Figures 1C to 1H. In total it took 83 and 148 sec to complete the oviposition behavior from the appearance to disappearance of the ovipositor inside the cavity in bamboo internodes. Abbreviation: o, the distal part of ovipositor.

Females of the lizard beetle *Doubledaya bucculenta* Lewis (Coleoptera: Erotylidae: Languriinae) have a large asymmetric head with enlarged left mandible and elongated forelegs [24] (Figure 1A). In spring, they excavate a small hole on a recently-dead culm of *Pleioblastus* and *Semiarundinaria* bamboos and insert the ovipositor into the internode cavity through the hole [24–27] (Figure 1B). They deposit an egg on the inner surface of a substantially sterile (i.e. fungus-free) cavity of the bamboo internode [15]. Doing so, they also inoculate the specific Saccharomycetes yeast *Wickerhamomyces anomalus* (E.C. Hansen) Kurtzman, Robnett & Bas.-Powers, which is retained in a mycangium adjacent to the ovipositor, inside the internode [15]. After that, they plug the hole with bamboo fibers [15,25]. Within the bamboo internode, the yeast propagates exclusively and a single larva of *D. bucculenta* develops on the yeast [15]. The insects overwinter at larval and adult stages. Adult beetles excavate the bamboo wall to make an exit hole and emerge in spring.

 The aims of this study were to determine whether *D. bucculenta* females inoculate the yeast onto the eggs, bamboo internodes, or both, and whether or not the larvae disperse yeast cells all over the inner surface of internode to facilitate yeast proliferation. Finally, we discuss the difference in the symbiont transmission and garden making strategies between social and non-social organisms.

## Materials and Methods

### Insects and bamboos

 Almost all insect samples of *D. bucculenta* and internode samples of the bamboo *Pleioblastus simonii* were collected in Kawaminami Town, Miyazaki Prefecture, Japan [32°9’N, 131°29’E] in May 2012 and April and May 2013 and in Hitachi City, Ibaraki Prefecture, Japan [36°38’N, 140°33’E] on 12 and 19 June 2012. Two other eggs were collected from *P. simonii* internodes in Shirosato Town, Ibaraki Prefecture, Japan [36°26’N, 140°21’E] on 12 June 2012. The relatively limited sample size of the beetles was due to extremely low densities of *D. bucculenta* [25]. No specific permits were required for the described field studies. The locations are not privately-owned or protected in any way. The field studies did not involve endangered or protected species.

### Behavior of *Doubledaya bucculenta* ovipositors inside the bamboo internode cavities

 Four adult females (11.56 mm to 14.21 mm in elytral length) and two adult males (8.08 mm and 8.63 mm in elytral length) of *D. bucculenta* were collected in Kawaminami in 2012. *Pleioblastus simonii* internodes were also collected at the same time. Two groups of two females and one male were placed separately in two transparent containers, 36 × 21 cm at the bottom, 40 × 24 cm at the top and 27 cm tall. The containers had five and eight internodes inclined on the walls. Females usually excavate small holes on bamboo internodes, insert the ovipositors into bamboo internodes through the holes, and deposit the eggs on the inner surface of internodes. Therefore, when a female was about to finish excavating a hole, the internode with the excavating female was taken out and carefully split longitudinally. The outer side of split internode was leaned on a corrugated plastic plate placed in the new container and the behavior of the ovipositors inside the internodes was recorded using a digital video camera HDR-XR350V (SONY, Tokyo, Japan). Observation was made at 25 °C.

### Fungal proliferation inside the bamboo internode cavities before larval hatch

Thirty adult females (6.57 mm to 16.05 mm in elytral length) and 26 adult males (6.85 mm to 15.16 mm in elytral length) of *D. bucculenta* and *P. simonii* internodes were collected in Kawaminami in 2013. Each group of three females and three males was placed in a transparent container, 37 × 22, and 24 cm tall. The containers had eight to ten internodes inclined on the walls. After three days, six oviposition mark-bearing internodes were split longitudinally. The inner surface of the internode was observed under a stereomicroscope. Rearing was made at around 25 °C.

### Localization of yeasts on *Doubledaya bucculenta* eggs and *Pleioblastus simonii* internodes

**Figure 2:**
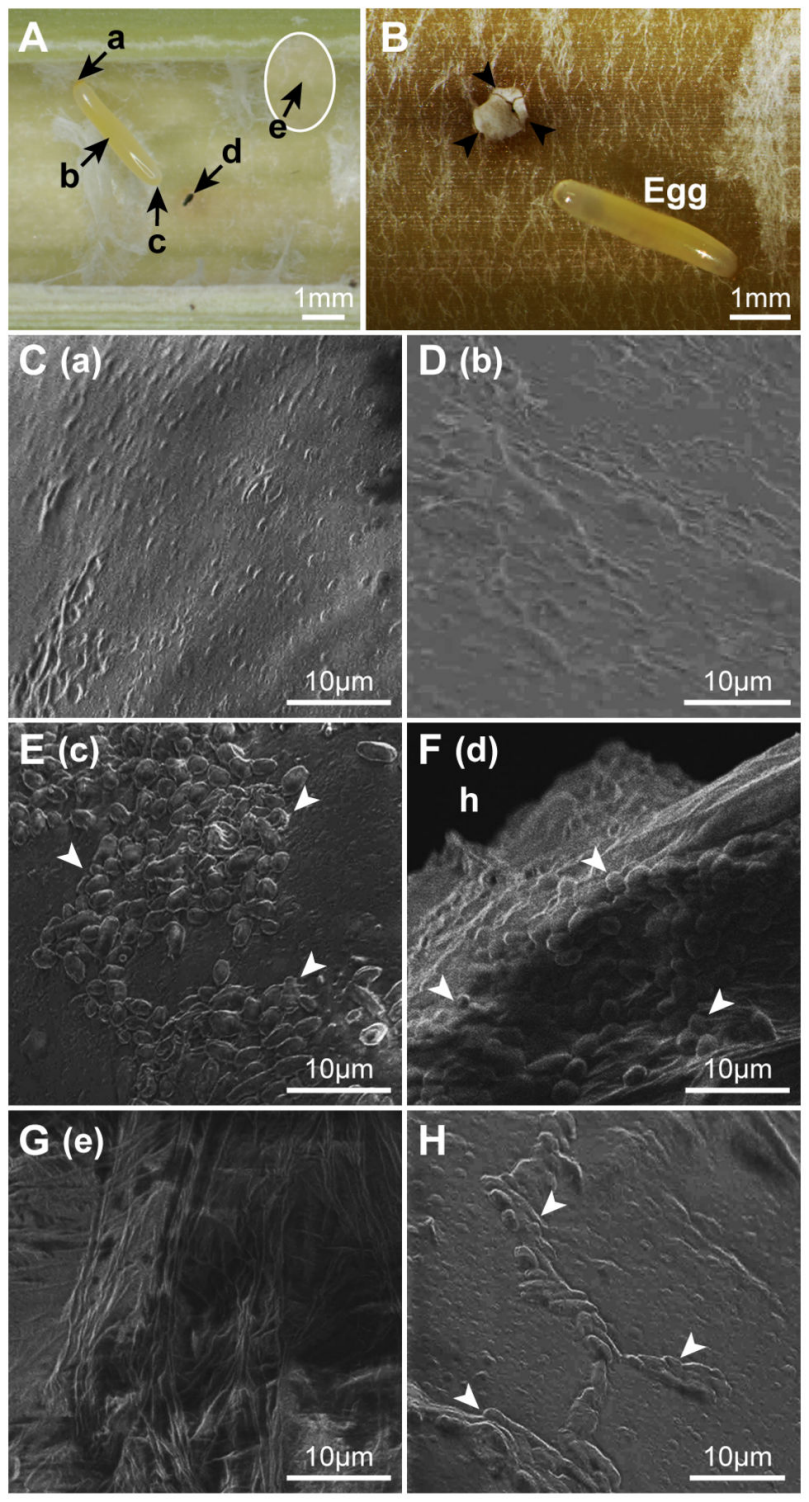
Symbiotic yeasts on *Doubledaya bucculenta* eggs and *Pleioblastus simonii* internodes. (A) An egg deposited on the inner surface of the internode. Black arrows a to e indicate sites whose cryo-SEM micrographs were taken and from which microbes were isolated. (B) Yeast colonies (black arrowheads) growing at the inner opening of *D. bucculenta* oviposition hole on a *P. simonii* internode. Yeast colonies are invisible on the egg surface. White to translucent pieces are fragmented dead pith tissues of *P. simonii*. (C to H) Cryo-SEM micrographs of (C) the anterior end of an egg (a in Figure A), which came out of the ovipositor first during oviposition and was attached to the inner surface of internode, (D) the lateral side of an egg (b in Figure A), (E) the posterior end of an egg (c in Figure A), which came out of the ovipositor finally during oviposition and was held in the air after oviposition, (F) the edge of the inner opening of an oviposition hole (d in Figure A). h-oviposition hole. (G) The inner surface of internode (e in Figure A) 1 cm distant from an oviposition hole, and (H) the anterior end of an egg when a mite was found at the inner opening of an oviposition hole. White arrowheads indicate the yeast cells.

 Ten dead bamboo internodes with fresh oviposition marks of *D. bucculenta* were collected in Hitachi. They were soon washed with running tap water and surface-sterilized with 70% ethanol. After the internodes were carefully cut open, a piece of sterilized wet filter paper (Quantitative Filters Papers No.5B, Toyo Roshi, Tokyo, Japan), 0.5 mm × 0.5 mm × 0.21 mm thick, was placed on one of five different places for one second; (a) near the anterior ends of eggs, (b) on the lateral sides of eggs, (c) at the posterior ends of eggs, (d) at inner openings of the oviposition holes, and (e) at inner surface of the internode 1 cm distant from the oviposition hole (Figure 2A). Pieces of filter paper were placed separately in 1.5 ml tubes containing 100 μl of sterilized water and the water was vortexed to generate microbe suspensions. These suspensions were diluted 5, 10 and 50-fold with sterilized water, and 50 μl of diluted suspension was spread over potato dextrose agar (PDA) (Difco, Detroit, MI, USA) plates (diameter 9 cm) containing rifampicin (Wako, Osaka, Japan) at a concentration of 20 μg/ml.

In addition, the posterior ends of five eggs (from four Kawaminami-originating *D. bucculenta* females collected in 2012 laid into bamboo internodes in laboratory and one egg obtained from a bamboo internode in Shirosato) were placed in contact with antibiotic-free PDA plates for one second. 

All plates were held at 25°C for 2 days. Then the number of colonies was counted to determine yeast cell densities.

### Cryo-scanning electron microscopy observation

 Four *P. simonii* dead bamboo internodes with a *D. bucculenta* egg were collected in Hitachi and subsequently held at −80°C for more than three weeks. Cryo-scanning electron microscopy (cryo-SEM) observation was made on the anterior and posterior ends of eggs, lateral sides of eggs, inner openings of the oviposition holes, and inner surface of the internode 1 cm distant from the oviposition hole, following manufacturer’s instruction using JSM-6390LV (JEOL, Tokyo, Japan). In addition, one egg that a Kawaminami-originating female deposited on 19 May 2012 was also observed using cryo-SEM. 

### DNA sequencing and phylogenetic analysis

DNA sequences in the D1/D2 domain of 26S rRNA and translation elongation factor-1α (EF-1α) genes were determined to identify *W. anomalus*-like yeast isolates obtained from *D. bucculenta* eggs and *P. simonii* internodes in this study. In addition, we determined the DNA sequences of the internal transcribed spacer (ITS) region and the 5.8S rRNA gene of another fungal isolate with a colony texture different from *W. anomalus*-like yeast using the primers ITS5 (5’-GGAAGTAAAAGTCGTAACAAGG-3’) and ITS4 (5’-TCCTCCGCTTATTGATATGC-3’; [28]). Preparation, polymerase chain reaction (PCR) and sequencing of yeast and fungal DNA samples were performed following Toki et al. [15]. Nucleotide sequence data reported in this study have been deposited to the DNA Data Bank of Japan with accession numbers AB774379-AB774443. Multiple alignments of the nucleotide sequences were generated using the program ClustalX 1.83 [29]. Molecular phylogenetic analyses were conducted by the neighbor-joining method using the program PAUP 4.0b10 [30] with 1000 bootstrap replicates.

### Larval hatch and larval effects on yeast dispersal

 To determine from what part of eggs *D. bucculenta* larvae emerge, microscopic observations were conducted with two and six eggs of *D. bucculenta* collected in Shirosato and Hitachi from *P. simonii* internodes, respectively. 

 In addition, to determine whether *D. bucculenta* larvae disperse the yeast cells in internode cavities, we made an experiment using yeast-free larvae and a *W. anomalus* isolate subcultured on PDA, which had been obtained from a *P. simonii* internode in Kawaminami [15]. Fourteen eggs of *D. bucculenta* were collected from recently-dead *P. simonii* internodes in Hitachi. Five of them were kept at −80°C for 21 days to kill them. Nine other eggs were kept at 10°C for 14 or 17 days. Then, the living and dead eggs were surface-sterilized in 99.5% ethanol for ten seconds and then in 70% ethanol for ten seconds to obtain yeast-free eggs [15]. After that, four of the nine living eggs were placed singly on PDA plates in 9-cm-diam. Petri dishes at 25°C to obtain the first instar larvae.

Yeast cell suspension was obtained by adding sterilized water onto the yeast colonies cultured on PDA plate. The suspension was diluted down to 10^6^ colony forming units (CFUs) ml^−1^ before use. Pieces of filter paper, 0.5 mm × 0.5 mm × 0.21 mm thick, were immersed in the yeast cell suspension and brought in short contact either with the center of PDA plates or the eggs.

We made four experimental groups. In the first group, yeast cells were inoculated on five PDA plates by contact with a piece of paper containing yeast cell suspension, but neither the eggs nor larvae were added. In the second group, four 3-day-old first instar larvae were placed singly on the edge of yeast-inoculated PDA plates. In the third group, five yeast-inoculated dead eggs were placed singly on an autoclaved strip of a *P. simonii* internode (about 15 cm × 1.6 cm in size) in sterilized test tubes (3.0 cm in diameter and 20 cm tall) with moistened cotton placed at the bottom. In the fourth group, five yeast-inoculated living eggs were placed singly on an autoclaved strip of a *P. simonii* internode (about 15 cm × 1.6 cm in size) in the aforementioned test tubes. Yeast cultures on PDA plates and internode strips were incubated at 25°C in the dark and observed daily to record the colony formation for three and four days, respectively.

### Statistical analysis

 Paired *t*-test and Shapiro-Wilk normality test were used to compare the number of yeast CFUs per mm^2^ between different sites of eggs and internode cavities, and to determine whether or not the differences in yeast densities showed normal distribution, respectively. Calculation was performed using R 2.15.1 [31].

## Results

### Behavior of *Doubledaya bucculenta* ovipositors inside the bamboo internode cavities

 Behavior of the ovipositor inside the bamboo internodes was observed for two females A (11.56 mm in elytral length) and B (14.21 mm in elytral length).

After females completed the excavation of the oviposition holes in the internode wall using their mandibles, they inserted the ovipositors into the internode cavities through the holes (Figure 1C, Video S1). After a while (28 to 115 sec, n = 2), an egg began to come out of the ovipositors (Figure 1D and I) and the anterior end of the egg was attached to the internode surface (Video S1) (3 to 6 sec, n = 2). When the egg came out of the ovipositor completely and the posterior end of the egg was in contact with the tip of ovipositor, the ovipositor of female A exhibited nine peristaltic movements for 24 seconds, and that of female B exhibited 10 peristaltic movements for 20 seconds (Figure 1E, F and I, Video S1). After detaching the ovipositor from the egg (Figure 1G and I), the female withdrew it (Figure 1H and I), and immediately plugged the oviposition hole with bamboo fibers (Video S1). It took 2 to 8 sec (n = 2) for ovipositors to be detached from an egg following the end of peristaltic movements and it took 2 to 13 sec (n = 2) to be withdrawn after being detached from the eggs.

In total it took female A 83 sec and female B 148 sec to complete the oviposition behavior, which was observed during a period from the appearance to disappearance of the ovipositor inside the cavity of bamboo internodes (Figure 1I).

### Fungal proliferation inside the bamboo internode cavities before larval hatch

 Microscopic observation of six *D. bucculenta* egg-bearing internodes revealed that white fungal colonies were visible on four of six openings of the oviposition holes within three days of oviposition, while no visible fungal colonies appeared on eggs or internode surface (Figure 2B).

### Localization of yeasts on *Doubledaya bucculenta* eggs and *Pleioblastus simonii* internodes

Microbial isolations revealed that *W. anomalus*-like yeast cells were always present at the posterior end of eggs and the inner openings of the oviposition holes (Table 1). The yeast cells were more abundant at the openings of oviposition holes than at the posterior end of eggs (Shapiro-Wilk normality test: *W* = 0.926, *P* = 0.412; paired *t* test: *t*_9_ = 4.032, *P* = 0.003, n = 10) (Table 1) (Figure 2B). The density of yeast cells per mm^2^ was much smaller near the anterior ends of eggs and on the lateral sides than on the posterior ends of eggs (Table 1). No yeast cells were detected on the inner surface of the internode 1 cm distant from the oviposition hole (Table 1). A white, matte fungal colony, which was different from the abovementioned yeast colonies, occurred on a PDA plate when microbes were sampled from the opening of oviposition hole on an internode.

**Table 1:** Localization of the symbiotic yeast *Wickerhamomyces anomalus* on *Doubledaya bucculenta* eggs and internode cavities of *Pleioblastus simonii* bamboo.

		Density of yeast cells (CFU/mm^2^)			
Isolation sites^a^	No. of eggs or internodes examined	Mean ± SD	Min	Max	No. of eggs or internodes having yeast cells
Near anterior ends of eggs	10	2.6 × 10^2^ ± 4.2 × 10^2^	0	1.2 × 10^3^	4
Lateral sides of eggs	10	3.2 × 10 ± 7.5 × 10	0	4.0 × 10^2^	3
Posterior ends of eggs	10	7.3 × 10^3^ ± 3.4 × 10^3^	1.3 × 10^3^	1.6 × 10^4^	10
Inner openings of oviposition holes	10	4.8 × 10^4^ ± 3.3 × 10^4^	4.4 × 10^3^	1.3 × 10^5^	10
Inner surface of the internode 1 cm away from oviposition holes	10	0 ± 0	0	0	0

a Microbial isolation was made from three different places of eggs and two different places of internodes harboring the eggs.

Instantaneously pressing the posterior ends of one and four eggs derived from two other locations, Shirosato and Kawaminami, respectively, onto PDA plates caused *W. anomalus*-like yeast colonies to occur.

### Cryo-scanning electron microscopy observation

 Yeast cells were localized at the posterior ends of three eggs and the edges of the inner openings of three oviposition holes in the internodes, whereas no yeast cells were found on the anterior ends and lateral sides of the three eggs and the inner surface of the internode 1 cm distant from the three oviposition holes (Figure 2C–G), when no mites were observed. When a mite of the genus *Trichouropoda* (Uropodidae) was found at the inner opening of an oviposition hole, yeast cells were sparsely and widely distributed on the anterior and posterior ends of the egg (Figure 2H).

Cryo-SEM observation also revealed that a Kawaminami-originating egg had yeast cells on the posterior end but did not have them on the anterior end or the lateral side.

### DNA sequencing and phylogenetic analysis

**Figure 3:**
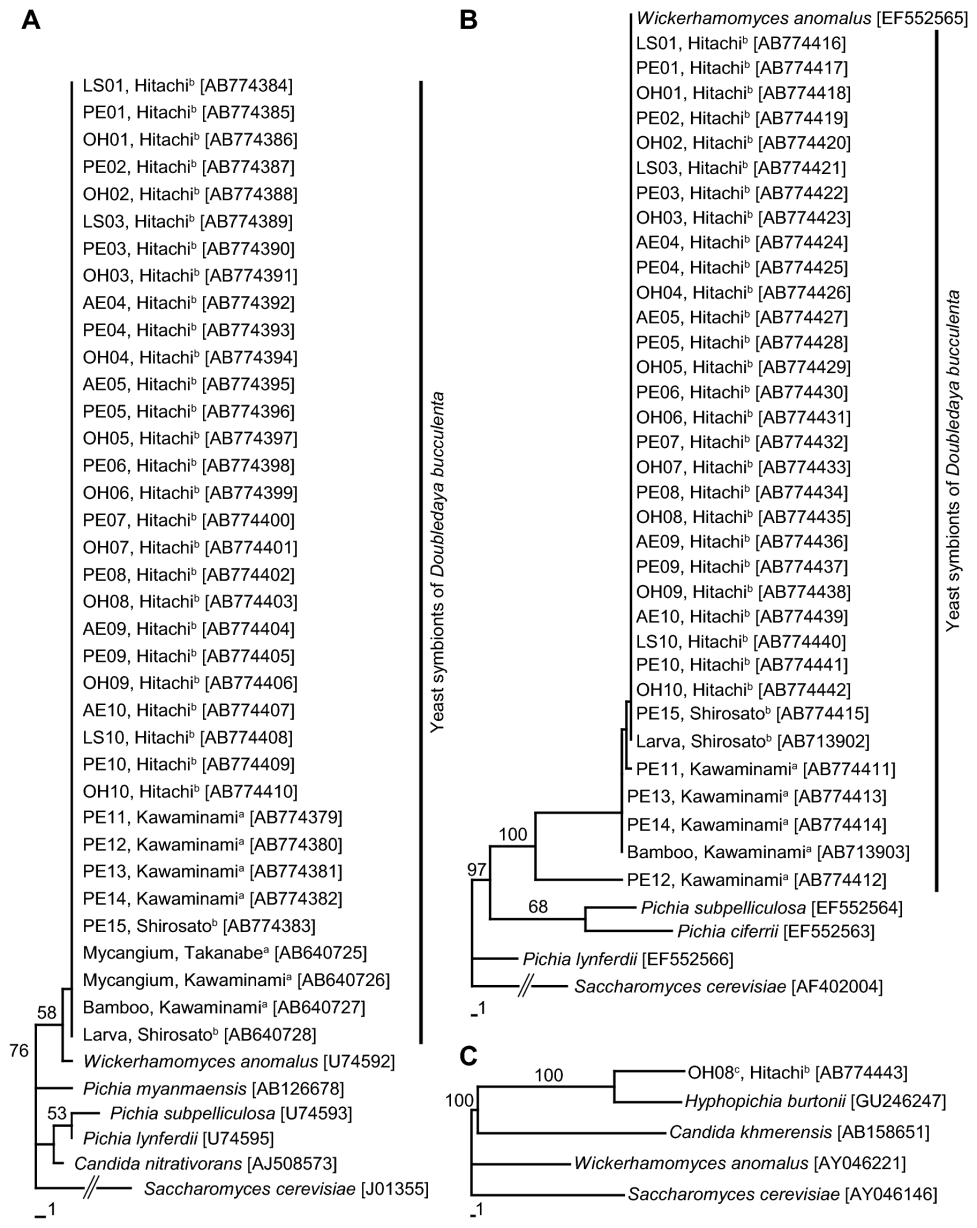
Phylogenetic placement of the yeast isolates associated with *Doubledaya bucculenta*. (A) A neighbor-joining phylogeny inferred from DNA sequences of D1/D2 domain of 26S rRNA gene (530 bps). (B) A neighbor-joining phylogeny inferred from DNA sequences of EF-1α gene (709 bps). (C) A neighbor-joining phylogeny inferred from DNA sequences of ITS/5.8S rRNA gene (360 bps). Bootstrap values (1000 replicates) of 50% or higher are shown at the nodes. Sequence accession numbers are shown in brackets. Yeast isolates obtained from *D. bucculenta* and *Pleioblastus simonii* in this study are discriminated by sites of eggs and *P. simonii* internodes where the isolate was obtained, the number to specify an egg and the internode harboring the egg, and the localities where the isolates were sampled. Abbreviations: AE, the anterior end of an egg; LS, the lateral side of an egg; PE, the posterior end of an egg; OH, the edge of the inner opening of an oviposition hole. ^a^Miyazaki Prefecture, Japan; ^b^Ibaraki Prefecture, Japan; ^c^a yeast isolate with colony texture different from *Wickerhamomyces anomalus*, which was obtained at the inner opening of an oviposition hole.

 A 0.5-kb fragment of 26S rRNA gene and a 0.8-kb fragment of EF-1α gene were amplified by PCR and sequenced for 32 *W. anomalus*-like yeast isolates; four from the anterior ends of eggs, three from the lateral sides of eggs, ten from the posterior ends of eggs, and ten from the inner openings of oviposition holes on internodes, which were collected in Hitachi, and one and four from the posterior ends of eggs collected in Shirosato and Kawaminami, respectively. The 530 bp sequence of the 26S rRNA gene was identical between yeast isolates examined in this study and *D. bucculenta*-originating yeast isolates reported so far (accession numbers AB640725-AB640727; [15]). The sequence was also identical to that of *Wickerhamomyces anomalus* (accession number U74592; [32]) (Figure 3A). Similarly, the 709 bp sequence of EF-1α gene was identical between yeast isolates in this study and *D. bucculenta*-originating yeast isolates reported (accession numbers AB713902, AB713903; [15]) except for five nucleotide sites with ambiguous base reading. The sequence was also identical to that of *W. anomalus* (accession number EF552565; [33]) except for six nucleotide sites with ambiguous base reading (Figure 3B).

 A 0.4-kb fragment of ITS/5.8S rRNA gene was amplified by PCR and sequenced for the other fungal isolate obtained at an opening of the oviposition hole sampled in Hitachi. The fungal isolate had the 360 bp sequence closely similar (98%) to the Saccharomycetes yeast *Hyphopichia burtonii* (= *Pichia burtonii*, accession number GU246247; [34]), which is phylogenetically far from *W. anomalus* [35] (Figure 3C). The sequence showed a low similarity of 71% between the fungal isolate and *W. anomalus* (accession number AY046221; [36]).

### Larval hatch and larval effects on yeast dispersal

 Microscopic observation of eight *D. bucculenta* eggs revealed that all larval heads appeared at the posterior ends of eggs and the larvae came out of there.

**Figure 4:**
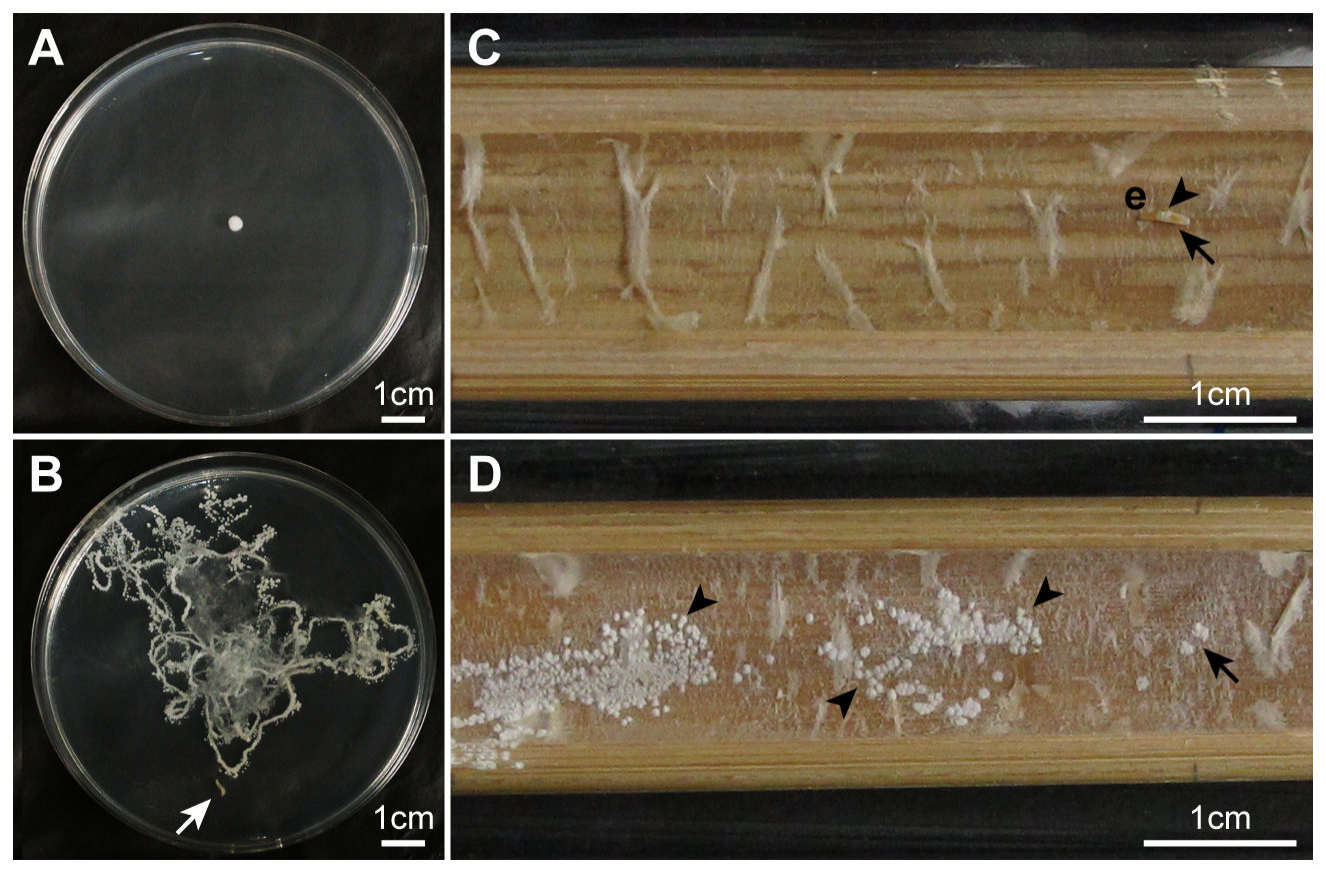
Effects of *Doubledaya bucculenta* larvae on the dispersal of *Wickerhamomyces anomalus* yeast cells. (A) A *W. anomalus* colony formed by the cells that were inoculated in the center of a potato dextrose agar (PDA) plate in the absence of a *D. bucculenta* larva. (B) *W. anomalus* colonies formed by the cells that were inoculated in the center of a PDA plate in the presence of a *D. bucculenta* larva. (C) No *W. anomalus* colony formation on an autoclaved *Pleioblastus simonii* internode strip where a yeast-inoculated dead egg of *D. bucculenta* was placed. (D) *W. anomalus* colonies formed on an autoclaved *P. simonii* internode strip where a yeast-inoculated living egg of *D. bucculenta* was placed. PDA Plates and internode strips were photographed three and four days after the incubation at 25 °C, respectively. White and black arrows indicate a first instar larva of *D. bucculenta* and a place where a yeast-inoculated egg of *D. bucculenta* was placed, respectively. Arrowheads indicate the yeast colonies. Abbreviation: e, egg.

 When the yeast cells alone were placed in the centers of five PDA plates, a yeast colony roundly expanded within a limited area in the center (mean ± standard deviation of colony diameter = 3.5 ± 0.3 mm, n = 5) in three days (Figure 4A). By contrast, when four first instar larvae (mean ± standard deviation of body mass of 3-day-old larvae = 0.5 ± 0.1 mg, n = 4) were individually placed on PDA plates with the yeast cells inoculated in the center, abundant yeast colonies occurred along the larval wandering tracks on three PDA plates in three days (mean ± standard deviation of body mass of 7-day-old larvae = 1.2 ± 0.4 mg, n = 3) (Figure 4B), whereas a small, round yeast colony occurred in the center of the remaining PDA plate because a released larva did not visit an area with yeast cells inoculated due to injured legs (colony diameter after three days, 3.1 mm; body mass of 7-day-old larva, 0.5 mg).

When the yeast-inoculated dead eggs were placed singly on the autoclaved strips of *P. simonii* internodes, a yeast colony expanded within a limited area around each egg (mean ± standard deviation of colony diameter = 2.0 ± 0.3 mm, n = 5) in four days (Figure 4C). By contrast, five yeast-inoculated living eggs produced the first instar larvae in one day and caused abundant yeast colonies to occur throughout the strips of *P. simonii* internodes in four days (Figure 4D).

## Discussion

This study demonstrated that (i) the symbiotic yeast cells were consistently present at the posterior ends of eggs and on the edges of the inner openings of *D. bucculenta* oviposition holes, (ii) the ovipositors of *D. bucculenta* showed peristaltic movement when they were in contact with the posterior ends of the eggs, (iii) newly hatched larvae, which came out of the posterior ends of eggs, acquired the yeast cells from the egg surface and dispersed them in the internode cavities, and (iv) the symbiotic yeast was spread much faster and wider on culture media and in the bamboo internode when a *D. bucculenta* larva was present than when it was absent. The results indicated that ovipositors of *D. bucculenta* would play a critical role in the vertical transmission of symbiotic yeast and that the larvae contribute actively to the establishment of a big fungal garden by facilitating fungal dispersal within internode cavities. To our knowledge, this study is the first to report the early process of symbiont transmission inside plants in non-social, plant-tissue boring insects that make fungal gardens, which was determined by experimental techniques and direct observation of ovipositors inserted into plants.

Yeast isolation and cryo-SEM observation showed that there were more abundant yeast cells at the inner openings of oviposition holes than at the posterior ends of eggs. This may suggest either the initial deposition of a larger amount of yeast cells on the edge of inner openings of oviposition holes than on the eggs or quicker propagation of yeast at the inner openings of the holes than on the egg surface, because the yeast propagates primarily on the inner surface of internode cavities as shown in an experiment of this study. By contrast, the density of yeast cells was much smaller on the anterior ends and lateral sides of eggs than on the posterior ends. 

*Doubledaya bucculenta* females carry the yeast cells in a pocket-like mycangium, which is the folded tergal integument of the eighth abdominal segment [15]. As the mycangium is not inserted into the bamboo internode cavity, it is likely that the ovipositor conveys the yeast cells into the internode cavity from the mycangium. Thus, peristaltic movement of the ovipositor may indicate not only inoculation of the yeast onto the posterior ends of the eggs but also transportation of the yeast from the outside of internode cavity to the inside via the ovipositor. The inner openings of oviposition holes are the narrowest parts of the cone-shaped holes and are much smaller in diameter than *D. bucculenta* eggs [15] (Figure 2A). Thus the relatively small oviposition holes may force the yeast mass to come out of ovipositor when the females retract the ovipositors, which may provide a presumable explanation of high yeast densities at the edge of oviposition holes and low yeast densities on the anterior ends and lateral sides of eggs: a small number of yeast cells remain on the inner wall and/or tip of ovipositor after the oviposition and are attached to the anterior ends and lateral sides of the next egg coming out of the ovipositor. 

Some plant-tissue boring insects carry symbiotic fungi in ovipositor-associated mycangia and transmit them in various ways. Some gall midge females of the tribe Asphondyliini insert the ovipositors into living plant buds several times. After each insertion, symbiotic fungal conidia were shed from the mycangia to a mixture of insect secretion and plant fluid exuding from drilled wounds [13]. The conidia would be transmitted into the oviposition channel when the females retract the ovipositors after egg deposition [13]. When the wood wasp *Sirex Noctilio* females oviposit into dying *Pinus radiata* trees, they drill two or more tunnels in wood via a single hole drilled in bark. They deposit the eggs in early made tunnels and the symbiotic fungus in the final tunnel [23]. In the case of a secondary wood borer, the lymexylid beetle *Hylecoetus dermestoides* females deposit the eggs on the outside of host trees. During the oviposition, they squeeze the symbiotic fungal spores out of the mycangia and make them adhere to the egg surface [10]. Newly hatched larvae contaminated with the spores bore into the wood and transmit the spores on the tunnel wall. The present study showed that *D. bucculenta* females deposit the symbiotic yeast on the eggs and on the inner surface of recently dead bamboo internode cavities away from eggs. Thus, when insects possess ovipositor-associated mycangia, the manner of fungal inoculation by ovipositors varies depending on the manner of oviposition, the physiological states of host plants, and responses of plant tissues to deposited eggs and inoculated fungi.

Social fungus-growing insects such as attine ants, macrotermitine termites, and xyleborine ambrosia beetles need to sequester fungal gardens from the environment to omit microbial contaminations [4]. Physical sequestering of fungal gardens is also observed in non-social fungus-growing insects such as lasiopterine and asphondyliine gall midges [13], leaf-rolling weevils of the genus *Euops* [11,12,14], the lymexylid beetle *H. dermestoides*, and siricid wood wasps [10,18]. In the case of *D. bucculenta*, the females plug the oviposition holes with bamboo fibers immediately after oviposition [15]. After yeast was deposited on the edges of inner openings of oviposition holes, it sometimes formed colonies to cover over the inner openings of oviposition holes before larval hatch (Figure 2B). Physical block of oviposition holes with bamboo fibers and the yeast colonies may construct a double-blocking system to sequester the internode cavities from natural enemies and competitors of *D. bucculenta* and microbes antagonistic to the yeast.

At the highest level of cultivation mutualism, adults of social fungus-farmers such as ants, termites and ambrosia beetles reinoculate the fungal cultivars on plant materials in their nests/tunnels [2–4,6]. On the other hand, there have been few reports on symbiont reinoculation in the gardens by non-social animals exhibiting primitive cultivation mutualisms. Though the marine snail *Littoraria irrorata* modifies substrate for fungal invasion and growth, it does not reinoculate the fungal associate [7]. Though the damselfish *Stegastes nigricans* eliminates weed algae in algal garden, it is unknown whether it reinoculates the symbiotic alga or not [8]. In the case of the lymexylid beetle *H. dermestoides*, larvae would transmit the symbiotic fungus on the wall of tunnels they make in wood [10], although there has been no experimental evidence for larval reinoculation of the fungi. By contrast, we demonstrated experimentally that *D. bucculenta* larvae dispersed the symbiotic yeast in the internode cavities, providing a novel case of reinoculation of symbionts.

Host organisms that consume the ectosymbionts as food have a risk of food shortage due to complete consumption of the ectosymbionts. That is more likely when the amount of food (=ectosymbiont) is relatively small. However, the host organisms must not consume all of the ectosymbionts for their survival and reproduction. The slime mold *Dictyostelium discoideum* farms bacterial symbionts and consumes them as food but does not eat all of them [9]. The leafcutter ant *Atta sexdens* queen does not feed on the symbiotic fungi but on trophic eggs during the establishment of a fungal garden [37]. Placing yeasts on two sites in bamboo internodes may help *D. bucculenta* larvae reduce the risk of food shortage, although mites may disturb the dispersion of yeast on beetle eggs.

Another yeast species was detected at an inner opening of oviposition hole together with *W. anomalus*, indicating that invasion of the garden by other fungi can occur, even though *D. bucculenta* sequesters the garden. Furthermore, the result suggests that the replacement of symbiotic yeasts may originate from the fungal contamination during oviposition of *D. bucculenta*, although *D. bucculenta* larvae show nutritionally obligate dependency on *W. anomalus* [15].

In conclusion, we found that *D. bucculenta* ovipositors were not just a duct to deposit the eggs but a tool to surely transmit the symbiotic fungus from an ovipositor-associated mycangium, and that the larvae actively dispersed the fungus throughout the bamboo internode cavity, resulting in rapid establishment of a large fungal garden. Our findings contribute to the understanding of cultivation mutualisms in non-social organisms.

## Supporting Information

Video S1 **Oviposition behavior of the ovipositor of a *Doubledaya bucculenta* female inside an internode cavity of *Pleioblastus simonii* bamboo.** Immediately after an egg comes out of the ovipositor completely, the ovipositor exhibits peristaltic movements with touching the posterior end of the egg with its tip.
(WMV)
